# PRRT plus holmium‐166‐SIRT (HEPAR PLuS) versus PRRT‐only in patients with metastatic neuroendocrine tumors: A propensity‐score matched analysis

**DOI:** 10.1111/jne.70034

**Published:** 2025-04-29

**Authors:** W. B. Veldhuis, T. Walter, D. M. V. de Vries‐Huizing, J. Theysohn, S. Barton, E. D. Ekkelenkamp, B. Lachachi, R. J. G. de Jong, L. W. van Golen, H. Lanzafame, L. Milot, H. Lahner, M. E. G. H. Lam, M. E. T. Tesselaar, A. J. A. T. Braat

**Affiliations:** ^1^ Department of Radiology and Nuclear Medicine University Medical Center Utrecht Utrecht The Netherlands; ^2^ Department of Oncology Hospices Civils de Lyon Lyon France; ^3^ Department of Nuclear Medicine Netherlands Cancer Institute Amsterdam The Netherlands; ^4^ Department of Diagnostic and Interventional Radiology and Neuroradiology University Hospital Essen, University Duisburg‐Essen Essen Germany; ^5^ Barton Biostatistics Ltd Essex UK; ^6^ Medical Affairs Interventional Oncology Terumo Europe NV Leuven Belgium; ^7^ Department of Nuclear Medicine Hospices Civils de Lyon Lyon France; ^8^ Clinic for Nuclear Medicine University Hospital Essen, University Duisburg‐Essen Essen Germany; ^9^ Department of Radiology Hospices Civils de Lyon Lyon France; ^10^ Department of Endocrinology and Metabolism Division of Laboratory Research, University Hospital Essen, University Duisburg‐Essen Essen Germany; ^11^ Department of Gastrointestinal and Medical Oncology Netherlands Cancer Institute Amsterdam The Netherlands

**Keywords:** ^166^Ho‐SIRT, [^177^Lu]Lu‐DOTATATE, neuroendocrine tumor, PRRT, radioembolization

## Abstract

Patients with bulky neuroendocrine liver metastases (NELM) undergoing PRRT with [^177^Lu]Lu‐DOTATATE have a worse survival than patients with limited liver metastases. Previously, the safety and efficacy of additional selective internal radiotherapy (SIRT), using holmium‐166 (^166^Ho)‐microspheres, directly following PRRT in patients with NELM were confirmed in the prospective HEPAR PLuS study. The aim of the current study was to provide insight into the efficacy and survival benefit of PRRT + ^166^Ho‐SIRT over PRRT‐only by means of a propensity score matched historical cohort. A multicenter retrospective data collection was performed to match patients treated with PRRT‐only to the prospectively collected HEPAR PLuS study patients. Demographic, clinical, laboratory, and imaging data were collected. The primary endpoint was the proportion of patients with progression‐free survival (PFS) at 2 years after the start of PRRT. Secondary endpoints included the proportion of patients with 2‐year hepatic PFS (hPFS), general PFS and hPFS, objective response rates (ORR), and overall survival (OS). Twenty‐four patients were 1:1 matched and included in the analysis. All key matching criteria were balanced between cohorts if feasible. The proportion of patients with PFS and hPFS at 2 years was 68% and 82% after PRRT + ^166^Ho‐SIRT versus 55% and 50% after PRRT only. Time to median PFS was comparable (31 vs. 30 months). An initial delay in hepatic progression or death of any cause was observed in PRRT + ^166^Ho‐SIRT mNET patients (75% probability of PFS at 27 vs. 22 months), most notably in intestinal tumors (75% probability of PFS at 26 vs. 15 months). Best ORR was 71% after PRRT + ^166^Ho‐SIRT versus 25% after PRRT only. This study showed that ^166^Ho‐SIRT after PRRT (vs. PRRT‐only) had a positive effect on the liver disease progression in patients with NELM, increasing the 2‐year hPFS rate and tumor response and delaying hepatic progression or death. However, this effect did not translate into improving general PFS and OS.

## Graphical abstract

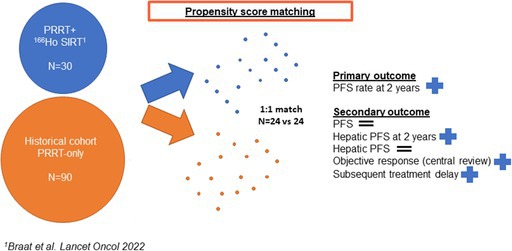

## 1 INTRODUCTION

After conclusion of the NETTER‐1 trial, peptide receptor radionuclide therapy (PRRT) with lutetium‐177‐DOTATATE ([^177^Lu]Lu‐DOTATATE; Lutathera®; Novartis) has become one of the standard second‐line treatments for gastroenteropancreatic neuroendocrine tumors (GEP‐NET), after failure of first‐line somatostatin analogues (SSA).^1^ In line with prospective and retrospective data, patients suffering from neuroendocrine liver metastases (NELM) have a poorer median overall survival (mOS) compared to those that do not. This is not only the case for PRRT, but for nearly all available therapies for patients with a NET.^2^, ^3^

In the post hoc analysis of the NETTER‐1 trial, liver tumor burden (fraction of liver involvement) was not predictive of a poorer survival in patients treated with PRRT.^4^ However, the extent of total disease burden in that study was limited (85% according to study's definition). Therefore, data and potential benefit for patients with more extensive liver disease could not be distilled from the NETTER‐1 trial. Earlier data from the Rotterdam group clearly demonstrated that both the presence of NELM (liver metastases 57 vs. no liver metastases 119 months mOS; HR 0.46, *p* < 0.01) and liver disease extent (extensive 46 vs. limited 123 months mOS; *p* < 0.01) were poor prognostic factors for OS.^5^ Furthermore, in the NETTER‐1 trial, patients having a “large lesion” (i.e., bulky disease), defined as having a target lesion >3 cm diameter, showed less benefit of PRRT in survival rate over patients without bulky disease.^4^ Interestingly, 75% (79/106) of the PRRT arm had bulky disease according to this definition, and additionally, of the considered target lesions in the bulky disease analysis, 70% were located in the liver. Objective response rate (ORR) 3 months after PRRT was limited (18%) according to Response Evaluation Criteria In Solid Tumors (RECIST 1.1), with no distinction reported between patients with/without bulky disease.^1^

Today, liver disease remains one of the biggest clinical challenges in the management of patients with metastatic NET, as it is difficult to treat. NELM are most commonly encountered as diffuse disease; subsequently, patients are not eligible for surgical resection and local ablative therapies, that is, radiofrequency ablation, and systemic therapies rarely result in tumor reduction. NELM are hypervascular and therefore induce preferential arterial flow. Due to these characteristics, trans‐arterial treatments have become more and more commonly applied in NELM. The ENETS and ESMO guidelines recognize all liver‐directed treatments, either as “debulking therapy” or as salvage therapy after failure of previous systemic therapies.^6^, ^7^ In particular, radioembolization (selective internal radiation therapy or SIRT) has gained interest due to high ORR, suggestion of longer progression‐free survival (PFS) over other liver‐directed therapies, and limited short‐term toxicities.^2^, ^8^ Recently, a prospective phase 2 study (HEPAR PLuS) demonstrated the safety and efficacy of additional holmium‐166‐microsphere radioembolization (^166^Ho‐radioembolization, or ^166^Ho‐SIRT) within 20 weeks following four cycles of 7.4 GBq PRRT in patients suffering from liver disease.^9^ HEPAR PLuS results were promising, showing high ORR (43%, according to RECIST 1.1), no significant loss in quality of life, and limited short‐term toxicities. However, the effect of the combination treatment (PRRT + ^166^Ho‐SIRT) on survival is unknown because no control arm was included. The aim of the current study was to gain insight into the efficacy and survival of PRRT + ^166^Ho‐SIRT over PRRT‐only in patients with bulky liver disease by performing a propensity score matched pair analysis using the data from the prospective HEPAR PLuS and a historical PRRT‐only cohort.

## 2 METHODS

### 2.1 Patient populations

For the PRRT + ^166^Ho‐SIRT cohort, data of the previously published HEPAR PLuS study was used for this analysis (*n* = 30).^9^ Participants of the HEPAR PLuS study provided written informed consent.

For the PRRT‐only cohort, a multicenter retrospective historical cohort was assembled from four European ENETS Centers of Excellence. In all participating centers for the PRRT‐only cohort, the need for informed consent for this retrospective cohort was waived by the local ethics committees. The treatment protocol for the PRRT‐only patients was part of the inclusion criteria.

Inclusion and exclusion criteria for the PRRT‐only cohort were matched to the criteria of the HEPAR PLuS study (Data S1). Follow‐up data included CT or MRI at 6, 9–12, 24 months, and later (if available) after the final cycle of PRRT. Data collection encompassed demographic, clinical, and imaging data (see Table S1 for more details).

### 2.2 Imaging data analysis

All CT/MRI imaging was blinded and centrally re‐assessed according to RECIST 1.1 for both cohorts. ORR at 3 months after the last PRRT and at subsequent follow‐up timepoints was collected. Additionally, baseline (i.e., pre‐PRRT), 3 months after the last PRRT, and 3 months after PRRT + ^166^Ho‐SIRT imaging were used to calculate fractional liver tumor involvement.

### 2.3 Endpoints

For all analyses and endpoints incorporating time, the start of the analysis was set on the date of the first cycle of PRRT (i.e., start of PRRT) in both cohorts. Endpoints incorporating a radiological response assessment (e.g., ORR) were all based on RECIST 1.1. A clear distinction was made between a patient‐based analysis (e.g., PFS), hepatic analysis (e.g., “hepatic PFS” = hPFS) and extrahepatic analysis.

The primary endpoint was defined as the proportion of patients with PFS at 2 years (PFS_2y_). Patients with radiological progression or death from any cause within 2 years were considered progressive. Patients who received subsequent treatment for NET (excluding SSA, and without radiological progression) within 2 years were included in the evaluation and considered progressive at the time of subsequent treatment. Patients with no response data available on or after 22 months (2 years minus 2 months) after the start of PRRT and with no recorded progression, death, or non‐SSA subsequent treatment within 2 years after the start of PRRT were considered lost to follow up for primary endpoint assessment.

Secondary endpoints included the proportion of patients with hepatic PFS at 2 years (hPFS_2y_; definitions in line with primary endpoint), hPFS in months, PFS in months, and OS. Patient‐based ORR and ORR of hepatic and extrahepatic disease were analyzed separately. Besides ORR at 3 ± 1 months after the last cycle of PRRT, the best ORR from 3 months after PRRT through the entire available follow‐up was reported.

Lastly, time to subsequent treatment was investigated, to establish whether additional therapy would extend the “treatment‐free” period of the patients.

### 2.4 Sample size and statistical analysis

A sample size of 120 patients was targeted (30 PRRT + ^166^Ho‐SIRT and 90 PRRT‐only) in order to perform a 1:*N* matching based on the greedy nearest neighbor method. The caliper width criterion was set at 0.25 times the standard error of the propensity score. Patients were matched based on their propensity score derived from a logistic regression model with treatment cohort as an outcome variable. The model was planned to include patient characteristics known to be predictors of outcome (see Data S2 for details). Matching was without replacement and the matching criteria were set such that, for each patient characteristic included in the propensity score model, the standardized mean differences were ≤±0.25 and the variance ratios were 0.5–2 between the two cohorts. Patients in either cohort with radiological progression at the 3‐month scan after the last cycle of PRRT were excluded for the Propensity Score Matched Analysis Set (MPA), that is, patients should have had the time to be considered for additional radioembolization, for adequate data comparison. Missing patient characteristic data was imputed.

A matching ratio of 1:1 was achieved, resulting in 24 patients per cohort. Any pre‐specified patient characteristics that were not included in the final propensity score model were also assessed for balance across the two cohorts on the final matched set. All patients enrolled in the study were included in the full analysis set (FAS) for analyses of baseline demographics, efficacy, and safety data. For analyses of PFS/PFS_*2y*_ and hPFS/hPFS_*2y*_, patients with progression (or hepatic progression for hPFS/hPFS_*2y*_) at 3 months after PRRT were excluded. The propensity score matched subset of patients was included in the MPA for further analyses of baseline demographics and efficacy data.

Kaplan–Meier time‐to‐event analyses were conducted. PFS and hPFS time‐to‐event analyses were based on all available central review scans up to 36 months after the start of PRRT. Patients were censored in case of subsequent treatment (excluding SSA). Overall survival time‐to‐event analyses were based on all available survival follow‐up data (approximately 3 years on average, but up to 7 years). Selected baseline demography, diagnosis characteristics, and previous treatments/resections tables, as well as selected efficacy tables and figures, are presented for MPA. FAS analyses are provided in Data S3. All analyses were repeated for different primary tumor groups: gastrointestinal NET (i.e., small intestine + colon + rectum) and pancreatic NET.

## 3 RESULTS

Data collection was performed between June 21, 2022 and March 31, 2023. Initiation of PRRT in the PRRT + ^166^Ho‐SIRT cohort was between January 2014 and December 2017, and in the PRRT‐only cohort between March 2014 and December 2019. Baseline characteristics for both the FAS and MPA of both cohorts are presented in Table 1.

**TABLE 1:** Baseline characteristics.

	Full analysis set		Matched analysis set	
	PRRT + ^166^Ho‐SIRT (*N* = 30)	PRRT only (*N* = 90)	PRRT + ^166^Ho‐SIRT (*N* = 24)	PRRT only (*N* = 24)
Demographics				
Mean age ± SD	62.0 ± 8.81	61.6 ± 10.25	62.5 ± 8.34	59.5 ± 10.50
Sex—Male	73%	52%	71%	67%
Sex—Female	27%	48%	29%	33%
Surgical resection				
Resection of primary NET	37%	63%	33%	42%
Hepatic metastectomy	0%	16.7%	0%	20.8%
Hepatic segmentectomy	6.7%	8.9%	4.2%	0%
Hepatectomy	3.3%	1.1%	4.2%	4.2%
Biliodigestive anastomosis	3.3%	1.1%	4.2%	0%
Cholecystectomy	6.7%	25.6%	4.2%	12.5%
Other metastasis resection	3.3%	4.4%	4.2%	12.5%
Other non‐NET resection	0%	12.2%	0%	12.5%
None	83.3%	57.8%	87.5%	62.5%
Prior treatments				
Prior SSA	70%	82.2%	70.8%	79.2%
Prior sunitinib/everolimus	13.3%	15.6%	12.5%	16.7%
Prior Bland embolization	6.7%	17.8%	8.3%	4.2%
Prior TACE	0%	3.3%	0%	0%
Prior chemotherapy	0%	21.1%	0%	33.3%
SIRT	0%	5.6%	0%	4.2%
Other	0%	8.9%	0%	16.7%
None	26.7%	2.2%	25%	0%
Primary tumor				
Small intestinal NET	40%	53%	46%	46%
Pancreatic NET	33%	29%	33%	29%
Bronchopulmonary NET	10%	2%	8%	4%
Colon NET	7%	3%	0%	4%
Rectal NET	7%	6%	8%	13%
Other	3%	7%	4%	4%
Tumor type				
Non‐functional	66.7%	55.6%	66.7%	66.7%
Functional	33.3%	44.4%	33.3%	33.3%
WHO grading				
NET Grade 1	40%	40%	37.5%	29.2%
NET Grade 2	60%	60%	62.5%	70.8%

KI67 index (%)	Mean ± SD (*N*)	Mean ± SD (*N*)	Mean ± SD (*N*)	Mean ± SD (*N*)
KI67 global index	6.0 ± 6.18 (29)	5.5 ± 4.88 (85)	6.4 ± 6.42 (23)	5.7 ± 4.91 (23)
KI67 primary tumor	3.1 ± 3.42 (12)	5.3 ± 4.96 (54)	3.4 ± 3.84 (9)	6.1 ± 4.99 (13)
KI67 liver metastases	8.7 ± 7.10 (19)	5.2 ± 4.29 (55)	9.1 ± 7.25 (16)	4.6 ± 4.40 (14)

Time in months since	Median (range)	Median (range)	Median (range)	Median (range)
First pathologic diagnosis	26 (2–193)	35 (1–374)	26 (2–193)	23 (1–100)
Liver metastases diagnosis	20 (2–88)	34 (1–288)	21 (2–88)	21 (1–96)
Liver tumor burden (%)	8 (1–81)	13 (0–83)	8 (1–81)	20 (0–64)

Three months after PRRT, that is, prior to any opportunity to receive ^166^Ho‐SIRT, four (13%) patients in the PRRT + ^166^Ho‐SIRT cohort and 16 (18%) patients in the PRRT‐only cohort had progressive disease by RECIST 1.1 (Table 2). These patients were excluded from the evaluation of PFS. Extrahepatic disease at 3 months post‐PRRT was more often observed in the PRRT + ^166^Ho‐SIRT cohort versus the PRRT‐only cohort (83% vs. 64%) in FAS. This imbalance was resolved in the MPA (88% in both arms; Table 2).

**TABLE 2:** PRRT details and imaging results 3 months after PRRT (comparison to FAS in Table S3).

Label	Matched analysis set	
	PRRT + ^166^Ho‐SIRT (*N* = 24)	PRRT only (*N* = 24)
	Mean ± SD	Mean ± SD
PRRT		
Total duration of PRRT (months)	6.61 ± 1.05	6.15 ± 0.89
Cumulative activity of PRRT (GBq)	29.68 ± 0.27	29.67 ± 1.01
Imaging results 3 months after PRRT		
Median liver tumor burden in % (range)	6 (1–84)	13 (0–69)
Liver tumor distribution		
Unilobar	8.3%	4.2%
Bilobar	91.7%	95.8%
Pattern of liver disease		
Simple	4.2%	0%
Complex	4.2%	8.3%
Diffuse	91.7%	91.7%
Objective response after PRRT alone^a^		
Partial response	25%	17%
Stable disease	75%	83%
Progressive disease	0%	0%
Presence of extrahepatic disease		
Extrahepatic disease	87.5%	87.5%
Lymph nodes	58.3%	58.3%
Bones	12.5%	37.5%
Lungs	25%	8.3%
Pleuritis	0%	0%
Peritonitis	4.2%	0%
Other	33.3%	33.3%

^a^ Prior to additional ^166^Ho‐SIRT.

**FIGURE 1:**
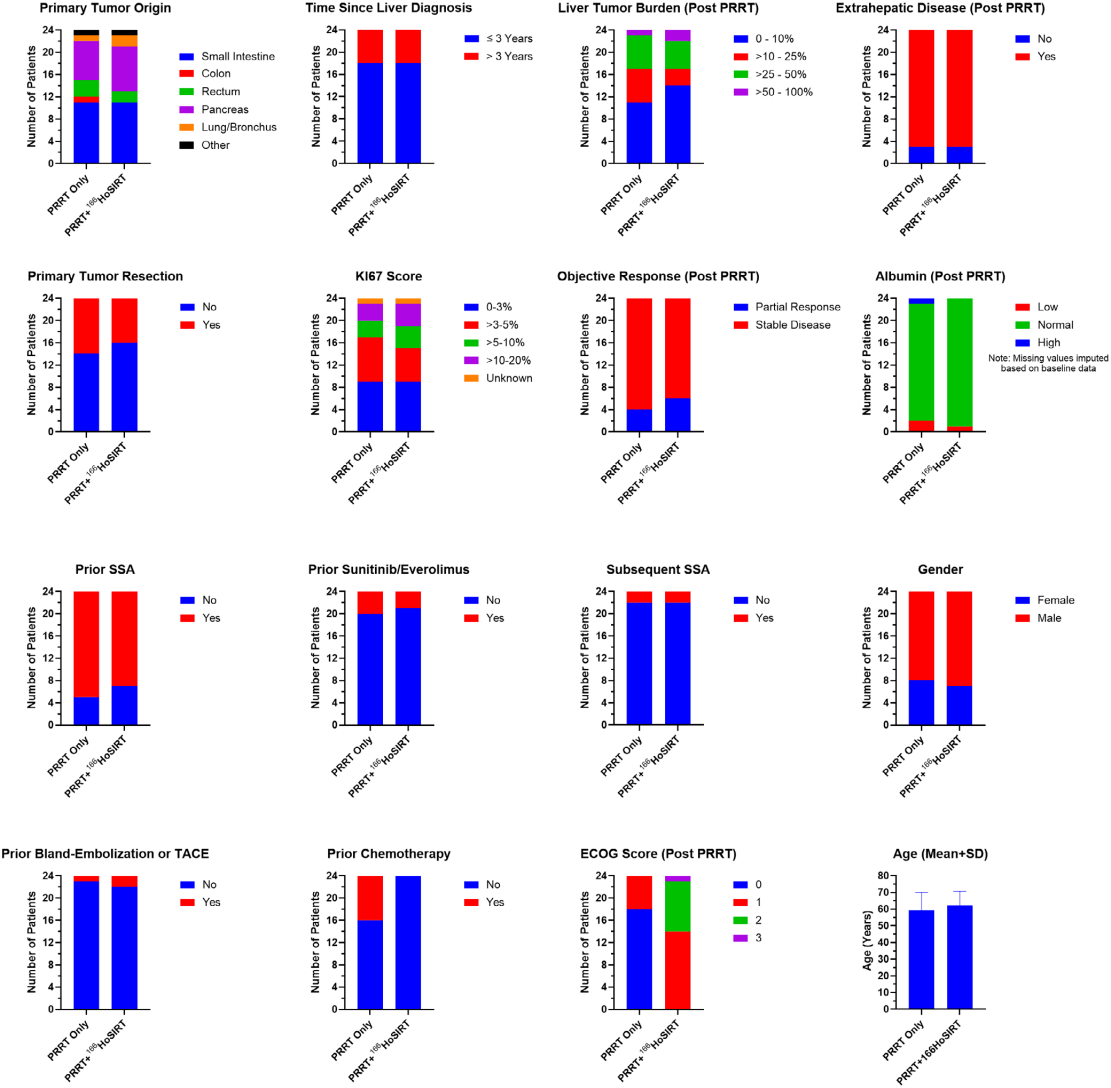
Patient characteristic matching parameters in the matched pair analysis.

Two matching criteria were severely imbalanced in the FAS and unable to be included in the final propensity score model, and hence residual imbalance remained in the MPA subset. Firstly, ECOG score at 3 months post‐PRRT (prior to ^166^Ho‐SIRT) showed an imbalance between cohorts, showing an approximate shift of +1 from pre‐PRRT scores in the PRRT + ^166^Ho‐SIRT cohort compared to the PRRT‐only cohort. Of note, ECOG scores were comparable across both treatment arms prior to PRRT (Table 3). Secondly, in the PRRT + ^166^Ho‐SIRT cohort, none of the patients received previous lines of chemotherapy, whereas 21% in the PRRT‐only cohort received chemotherapy. Other matching parameters were balanced in the MPA subset (Figure 1). Finally, the patient characteristics included in the final propensity score model as covariates are summarized in Table 4.

**TABLE 3:** Differences in Eastern Cooperative Oncology Group Performance scores (comparison to FAS in Table S4).

	Matched analysis set	
	PRRT + ^166^Ho‐SIRT (*N* = 24)	PRRT only (*N* = 24)
ECOG before PRRT		
0	50%	58.3%
1	45.8%	37.5%
2	4.2%	4.2%
ECOG 3 months after PRRT alone^a^		
0	0%	75%
1	58.3%	25%
2	37.5%	0%
3	4.2%	0%

^a^ Prior to additional ^166^Ho‐SIRT.

**TABLE 4:** Matching covariates included in final propensity score model.

Time from first liver diagnosis to start of PRRT
Previous primary tumor resection
Primary tumor origin
Ki67‐index pre‐PRRT
Fractional liver involvement at 3 months post‐PRRT
Prior somatostatin analogues
Extrahepatic disease at 3 months post‐PRRT
Prior sunitinib or everolimus
Prior bland‐embolization or chemoembolization
Sex

### 3.1 Progression‐free survival and overall survival analyses

Primary endpoint, PFS_2y_ was 68% (95% CI 43–87) in the PRRT + ^166^Ho‐SIRT cohort versus 55% (95% CI 32–76) in the PRRT‐only cohort. A total of 13 (54%) patients in the ^166^Ho‐SIRT + PRRT cohort and 15 (63%) patients in the PRRT‐only cohort had gastrointestinal NET. In the gastrointestinal subgroup, PFS_2y_ difference was more profound; PRRT + ^166^Ho‐SIRT 90% (95% CI 55–100; *n* = 10) versus PRRT‐only 57% (95% CI 29–82; *n* = 14). In the pancreatic NET subgroup, PFS_2y_ was comparable between groups (*n* = 6 in PRRT + ^166^Ho‐SIRT cohort and *n* = 7 in PRRT‐only cohort).

Secondary endpoints included hPFS_2y_, hPFS, and PFS. hPFS_2y_ was higher for PRRT + ^166^Ho‐SIRT, and differences were larger than those for PFS_2y_: 82% (95% CI 57–96) with PRRT + ^166^Ho‐SIRT versus 50% (95% CI 28–72) with PRRT‐only. Again, the difference in hPFS_2y_ was more profound in the gastrointestinal subgroup: PRRT + ^166^Ho‐SIRT 90% (95% CI 52–100; *n* = 9) versus PRRT‐only 50% (95% CI 23–77; *n* = 14).

**FIGURE 2:**
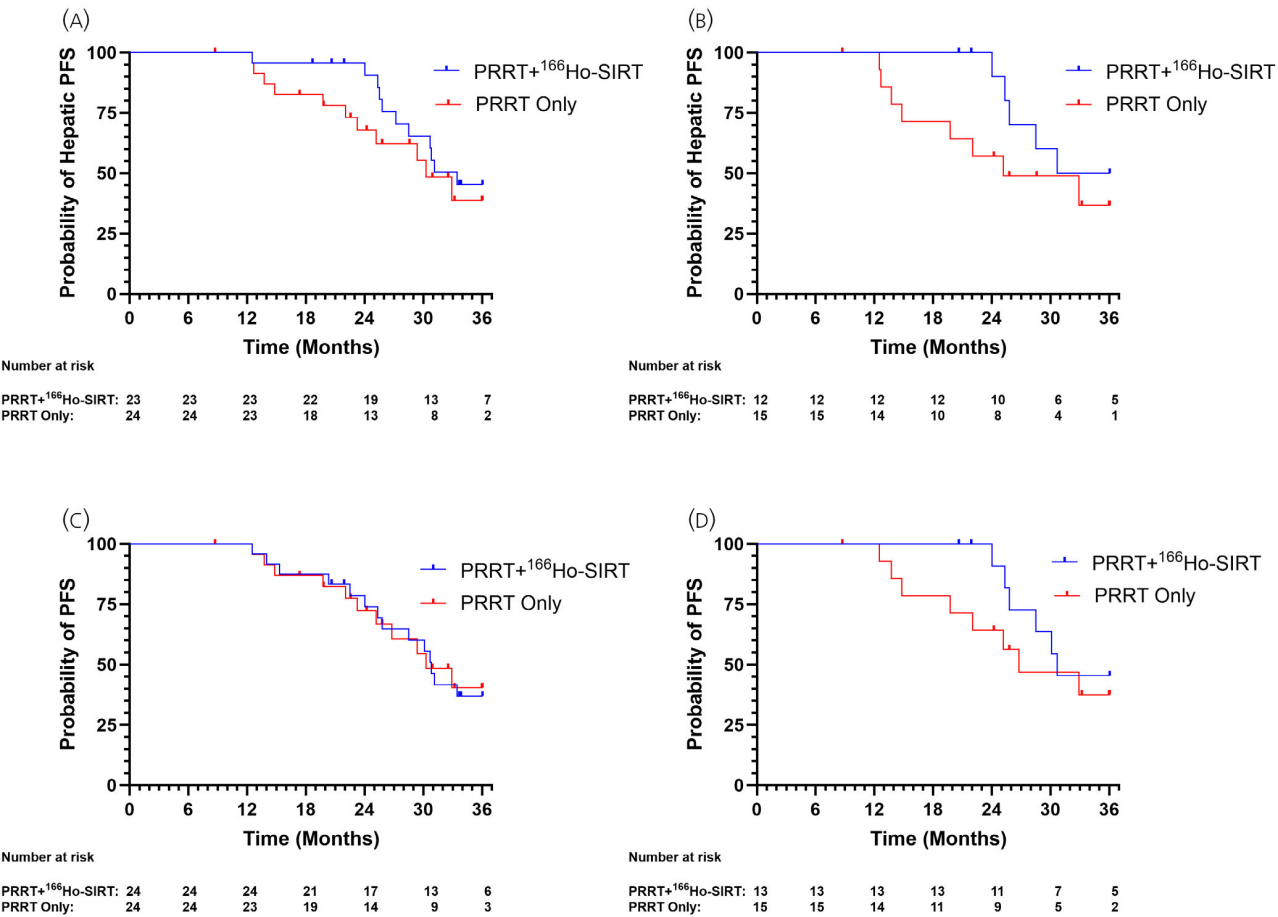
Kaplan–Meier analysis on PFS and hepatic PFS. (A) Hepatic PFS (hPFS), (B) hPFS in a subgroup of gastrointestinal NET, (C) progression‐free survival (PFS), (D) PFS in the subgroup of gastrointestinal NET.

The effect of a higher hPFS_2y_ can be seen in the hPFS Kaplan–Meier analysis, which showed a delay in hepatic progression or death (Figure 2A), while median hPFS was comparable: 33.5 months (95% CI 27.2–NE) in the PRRT + ^166^Ho‐SIRT cohort and 30.3 months (95% CI 22.0–NE) in the PRRT‐only cohort. In the gastrointestinal subgroup, a temporary delay in time to hepatic progression or death in the PRRT + ^166^Ho‐SIRT cohort was also observed (Figure 2B).

Median PFS showed no difference; 30.8 months (95% CI: 25.4–NE) in the PRRT + ^166^Ho‐SIRT cohort and 30.3 months (95% CI: 23.3–NE) in the PRRT‐only cohort (Figure 2C). In the gastrointestinal subgroup, however, a temporary delay in time to progression or death in the PRRT + ^166^Ho‐SIRT cohort was observed (Figure 2D).

**FIGURE 3:**
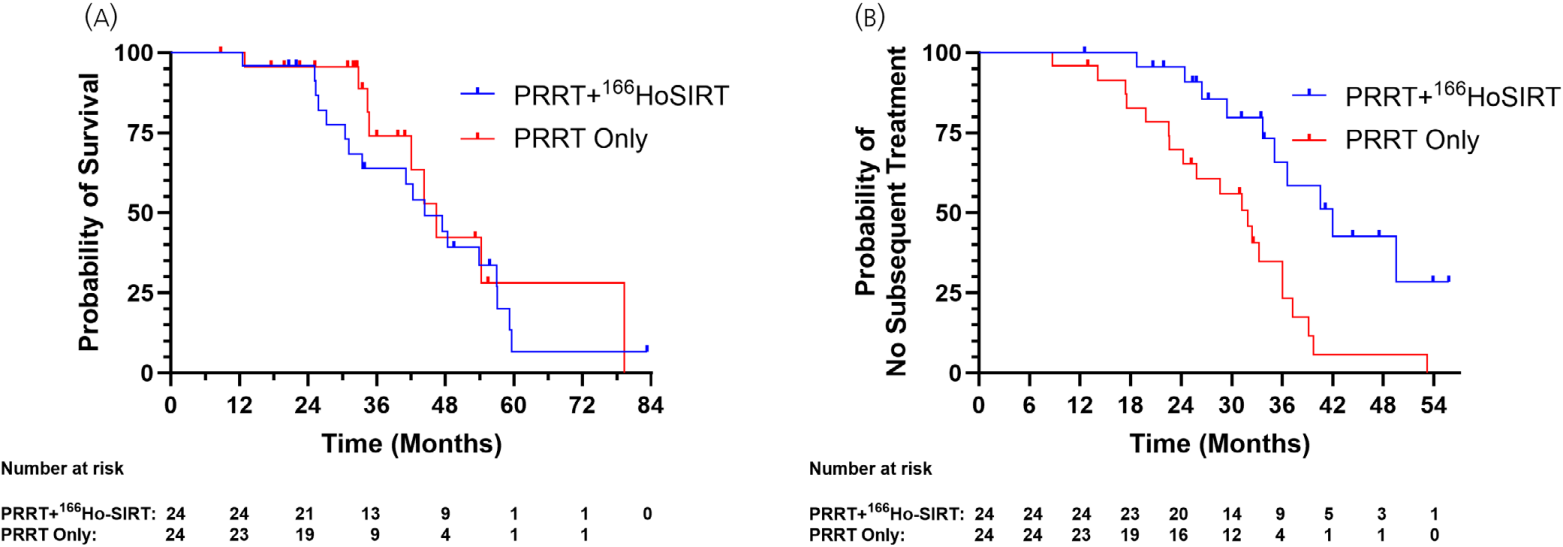
Kaplan–Meier analyses for overall survival (OS) and time to subsequent treatment. (A) Overall survival. (B) Time to subsequent treatment.

In post hoc Kaplan–Meier analyses, no OS benefit in patients receiving PRRT + ^166^Ho‐SIRT versus PRRT‐only was observed (Figure 3A), driven by a higher proportion of deaths observed in the PRRT + ^166^Ho‐SIRT cohort. Patients receiving subsequent treatment were not censored; however, a notably smaller proportion of patients received subsequent treatments after initial PRRT in the PRRT + ^166^Ho‐SIRT cohort versus the PRRT‐only cohort: 42% versus 87% (SIRT was not counted as subsequent treatment in the PRRT + ^166^Ho‐SIRT cohort).

### 3.2 Best objective response

Centrally reviewed best objective response data, based on available response assessments from 3 months after the end of PRRT onwards, is presented in Table S2. Overall, bORR was considerably higher in the PRRT + ^166^Ho‐SIRT cohort than the PRRT‐only cohort (71% vs. 25%); this was reflected by intrahepatic bORR (MPA: 75% vs. 29%). The localized hepatic effect of ^166^Ho‐SIRT is underscored by showing no improvement in bORR in the extrahepatic radiological response (17% vs. 13%).

### 3.3 Time to subsequent treatment

A delayed time to subsequent treatment (excluding SSA) was seen in the PRRT + ^166^Ho‐SIRT cohort (Figure 3B). Median time to subsequent treatment was 42.0 months (95% CI 33.7–NA) in the PRRT + ^166^Ho‐SIRT cohort and 31.9 months (95% CI 22.6–36.0) in the PRRT‐only cohort. Note, the PRRT + ^166^Ho‐SIRT cohort had a higher proportion of death events leading to an imbalance in censoring for this analysis. All subsequent treatments are noted in Table S5.

## 4 DISCUSSION

This propensity score matched analysis compared the prospective HEPAR PLuS study to a historical cohort of PRRT‐only patients, adhering to similar inclusion criteria. This propensity score matched analysis confirmed the added effect of ^166^Ho‐SIRT on top of PRRT on hepatic disease, as illustrated by the clinically significant improvement in hPFS_2y_ (82% vs. 50%), further supported by observed improvements in median hPFS (33.5 months, 95% CI 27.2–NE vs. 30.3 months, 95% CI 22.0–NE) and intrahepatic bORR (75% vs. 29%). These results are in line with the expectations of adding a liver‐directed therapy to a systemic disease. Interestingly, more profound differences are noticed in the gastrointestinal NET subgroup analysis for the same endpoints. For more generic endpoints, the PFS_2y_ analysis showed a trend towards improvement, but no significant improvement in PFS and OS was demonstrated.

Results from both cohorts in this study seem to be representative, with comparable PFS and OS rates to previously published studies. In the PRRT + ^166^Ho‐SIRT cohort, reported median PFS of 30.8 months and median OS of 44.3 months is comparable to reported results of three large retrospective series on SIRT‐alone. Notably, PFS is driven by extrahepatic disease progression in the entire PRRT + ^166^Ho‐SIRT cohort and also in the majority of SIRT‐alone studies.^3^, ^9^, ^10^, ^11^, ^12^ Regarding the PRRT‐only cohort, PFS was shorter than the reported PFS in NETTER‐1, but similar to the NETTER‐2.^13^ The PRRT‐only cohort often had WHO grade 2 disease (71% in current study) and tumor origin was heterogeneous. As for tumor reductive effect, bORR of both cohorts is similar to literature data.^1^, ^2^

The prospective HEPAR PLuS study focused on a real‐world, prognostically poorer patient population, and hence the prognosis of our real‐world historical cohort of PRRT‐only cohort was poorer by design compared to NETTER‐1. Additionally, compared to the PRRT‐only cohort, the PRRT + ^166^Ho‐SIRT cohort had more pulmonary metastases, known to have a negative influence on survival. However, fewer patients in the PRRT + ^166^Ho‐SIRT cohort had bone metastases, a negative prognosticator, compared to the PRRT‐only. More interestingly, all patients in the PRRT + ^166^Ho‐SIRT cohort had at least 1 ECOG point increase following PRRT (prior to ^166^Ho‐SIRT). This is striking and might be illustrative of a clinically poorer population than the PRRT‐only cohort (in which the majority remained ECOG 0). Being a retrospective comparative PRRT‐only cohort, data collection may have been less rigorous (i.e., suffering reporting bias); therefore, this observation should be considered with caution. However, having more extensive extrahepatic disease and potentially poorer ECOG scores, the PRRT + ^166^Ho‐SIRT cohort represents a poorer population than the current PRRT‐only cohort.

On the other hand, OS was similar to NETTER‐1 and the Rotterdam cohort for both the PRRT + ^166^Ho‐SIRT and PRRT‐only cohorts, even with the previously mentioned differences and assumed prognostically poorer populations.^1^, ^5^

Achieving high ORR with SIRT is well known, thus the superior bORR in the PRRT + ^166^Ho‐SIRT over PRRT‐only is not surprising. However, besides ORR, this MPA did demonstrate a clear hepatic PFS benefit or delay. This emphasizes the role of SIRT as a debulking therapy in patients suffering from bulky hepatic disease, as described in the most recent ESMO guideline of 2020.^7^ However, adhering to current guidelines, generally PRRT is initiated as a monotherapy.^6^, ^7^ Given the results of this study and previous published data, if PRRT is initiated to induce tumor reduction and effect is lacking, additional SIRT is a safe and feasible option; delaying hepatic PFS with a higher likelihood to induce hepatic tumor reduction. PRRT + ^166^Ho‐SIRT showed one of the highest overall responses in past NET literature on combination therapies. These results are comparable to combination therapies of capecitabine + temozolomide (CAPTEM) with either PRRT or SIRT in higher NET grades.^14^, ^15^ However, the specific patient population who may benefit most from direct initiation of CAPTEM with PRRT remains unclear and fear of additive hematological toxicities prevents adoption in clinical practice. For PRRT + CAPTEM, results of the CONTROL NET study are eagerly awaited.^16^ Combining PRRT + SIRT could be preferred over PRRT + CAPTEM, as added hematological toxicity is avoided (not restricting other subsequent systemic therapies) and initial effect of PRRT‐only can be observed first, without additional toxicities and SIRT can be employed as an “add‐on.”^15^, ^17^

With the observed improvement in hepatic PFS and ORR, the potential application of the combination may be most profound in liver‐only (bulky disease) patients, when assuming that treatment of liver disease will lead to PFS and quality‐of‐life benefit also. Unfortunately, in the current study, the number of patients with liver‐only disease was too limited to run a separate analysis. Therefore, no solid evidence was provided to support this hypothesis. But, as reported in the literature, liver‐directed treatments can control hormone‐related symptoms rapidly and delay the time to initiation of new treatment by PRRT + ^166^Ho‐SIRT, as suggested by this study.^3^ Hypothetically, prolonging a treatment‐free interval for patients (e.g., delaying initiation of systemic therapy) and with less hormone‐related complaints together may improve quality‐of‐life. Definitive evidence from a dedicated prospective study is needed to demonstrate this.

Another interesting finding in the current study was a more profound prolongation of survival or delay in progression in patients with NET from gastrointestinal origin, while no differences were noticed for pancreatic NET. Previous larger retrospective studies correlating tumor origin to SIRT outcomes have not reported this before.^1^, ^2^ Keeping the limited number of patients in mind (gastrointestinal NET *n* = 13 and 15, pancreatic NET *n* = 8 and 7, in the PRRT + ^166^Ho‐SIRT and PRRT‐only cohorts respectively), this finding warrants confirmation in future studies.

The results of this MPA are in support of a role for SIRT in the treatment of NET. However, the position of SIRT in the treatment paradigm of NET is still hindered by several unknowns. Firstly, there is no clear definition for “liver dominant” disease. The definition of liver‐only disease is clear and generally considered a good setting for liver‐directed therapy, for example, SIRT. In literature, guidelines and studies, no clear definition of “liver‐dominant” disease is described and in routine practice it is generally accepted to be intuitive. Unfortunately, this difficulty does not only apply to NET, but to many tumor entities. If we wish to clarify the role and best patients for SIRT (or any other locoregional therapy), this basic definition for NELM needs to be clarified to a certain extent. Secondly, concerns have been raised about long‐term hepatotoxicity, based on small retrospective studies, describing pseudo‐cirrhotic morphologies on imaging. Robust evidence related to this concern is limited, as most patients in reported studies initiated subsequent therapies after SIRT, leading to selection bias.^2^, ^18^, ^19^, ^20^, ^21^ Larger cohort studies report no issues for patients advancing to subsequent therapies, therefore the true clinical implications of the pseudo‐cirrhotic morphology on imaging remains unknown.^2^, ^3^, ^10^ Thirdly, short‐term quality of life is not hampered by SIRT, but long‐term quality‐of‐life changes remain unknown.^9^ Additionally, available data on SIRT in literature is all based on a “one size fits all approach” for all commercially available particles. SIRT in NET is running behind on recent developments in general SIRT practices, in which personalized dosimetry has become generally accepted. This approach has proven benefit for advanced hepatocellular carcinoma and has shown to be beneficial for metastatic colorectal carcinoma and cholangiocarcinoma.^22^, ^23^, ^24^ Recently for both ^90^Yttrium‐(glass) microspheres and ^166^Holmium‐microspheres, clear dose–response relationships have been demonstrated in NET as well (according to RECIST 1.1).^25^, ^26^ These retrospective analyses emphasize the added benefit of a patient‐tailored approach based on dosimetry. New prospective studies are needed to prove the added benefit of dosimetry‐based SIRT, to improve efficacy without increasing toxicity, as was shown for hepatocellular carcinoma previously.^22^

Additional limitations of the current study include the use of the prospective HEPAR PLuS study, compared to a retrospective PRRT‐only cohort. The PRRT‐only cohort suffered from reporting bias, which led to exclusion of toxicity analysis and effects on hormone‐related complaints in this study. Due to the heterogeneity of the primary tumors, propensity score matching was difficult, resulting in a 1:1 matching, and even with restrictive inclusion criteria and matching criteria, an imbalance in ECOG score remained. While reporting bias was present in the retrospective PRRT‐only cohort, important baseline characteristics (presence of extrahepatic disease, qualitative and quantitative liver tumor burden), reported results on hard endpoints (PFS, hPFS and OS) and reproducible ones (ORR) were all based on re‐assessment of imaging by independent central review. Therefore, results of the presented endpoints in the current study were minimized for various degrees of bias.

New prospective studies on SIRT in NET are needed and should focus on the timing and treatment sequencing with PRRT or other systemic therapies in different NET subtypes. More importantly, future studies should include prospective dosimetry and reporting of radiation metrics (compartment‐based mean absorbed dose, dose volume histogram metrics, etc.) to gain important data and insights into dose‐effect relationships and potential long‐term toxicities.

## 5 CONCLUSION

In this propensity score matched study, the combination of PRRT + ^166^Ho‐SIRT resulted in higher hepatic PFS rates at 2 years, median hepatic PFS, and bORR, compared to a historical PRRT‐only cohort, which was most profound in the gastrointestinal NET subgroup. No significant difference was demonstrated for median PFS and OS. The combination of PRRT + SIRT in NET may prove beneficial for patients with bulky liver‐only (or liver‐dominant) disease. Future prospective studies should investigate, elaborate, and confirm the findings of this propensity score matched study.

## CONFLICT OF INTEREST STATEMENT

The authors declare no conflicts of interest.

## Supporting information


**Data S1.** Supporting Information.

## Data Availability

The data that support the findings of this study are available from the corresponding author upon reasonable request.
