# Supplementary material for: PRRT plus holmium‐166‐SIRT (HEPAR PLuS) versus PRRT‐only in patients with metastatic neuroendocrine tumors: A propensity‐score matched analysis
Source: J Neuroendocrinol. 2025 Apr 29;37(8):e70034. doi: 10.1111/jne.70034 (PMC12358199; doi:10.1111/jne.70034)
Supplement: Supplementary file 1 — Data S1. Supporting Information. [file JNE-37-e70034-s001.docx]

#### **Supplemental data 1**

Inclusion criteria for the PRRT-only cohort were as follows:

- Well differentiated NET-patients of all origins.
- At least 3 measurable liver metastases (>1 cm longest diameter) at conclusion of PRRT.
- Completed 4 cycles of intravenous Lutetium-177-based PRRT with a cumulative dose between 26.00 and 32.56 GBq within a period of approximately 10 months.
- WHO performance status 0-2 at conclusion of PRRT.
- All PRRT cycles performed since 01-Jan-2014, with approximately up to 2 years of follow-up images available from the fourth PRRT treatment.
- Baseline and 3±1 month after PRRT anatomical imaging (CT and/or MRI) to be available and evaluable by central imaging review.

Exclusion criteria for the PRRT-only cohort were as follows:

- Grade 3 NET.
- Other than Lutetium-177-based PRRT.
- Bilirubin levels >21 µmol/L after PRRT.
- AST, ALT or alkaline phosphate >5x ULN after PRRT.

The PRRT+^166^Ho-SIRT cohort were part of the HEPAR PLuS study. Eligibility criteria for the HEPAR PLuS study were:

1. Patients must have given written informed consent.
2. Female or male aged 18 years and over.
3. Confirmed histological diagnosis NET, including bronchial carcinoids, and metastatic malignancy with liver metastases without standard therapeutic options for treatment including chemotherapy or surgery.
4. Patients must have been treated with 4 cycles of 200 mCi ^177^Lu-DOTATATE, the last cycle within 8-20 weeks of ^166^Ho-SIRT.
5. Life expectancy of 12 weeks or longer.
6. World Health Organisation (WHO) Performance status 0-2.
7. Liver disease with three or more measurable liver lesions according to the RECIST 1.1 criteria.
8. Negative pregnancy test for women of childbearing potential.

Exclusion criteria were:

1. Brain metastases or spinal cord compression, unless irradiated at least 4 weeks prior to the date of the experimental treatment and stable without steroid treatment for at least 1 week.
2. Radiation therapy within the last 4 weeks before the start of study therapy.
3. The last dose of prior chemotherapy has been received less than 4 weeks prior the start of study therapy.
4. Major surgery within 4 weeks or incompletely healed surgical incision before starting study therapy.
5. Any unresolved toxicity greater than National Cancer Institute (NCI), Common Terminology Criteria for Adverse Events grade 2 from previous anti-cancer therapy.
6. Serum bilirubin > 1.5 x Upper Limit of Normal (ULN).
7. Glomerular filtration rate <35 ml/min, determined according to the Modification of Diet in Renal Disease formula.
8. Alanine aminotransferase (ALT), aspartate aminotransferase (AST), or alkaline phosphatase (ALP) > 5 x ULN.
9. Leukocytes < 3.0 x 10^9^/l and/or platelet count < 100 x 10^9^/l.
10. Significant cardiac event (e.g. myocardial infarction, superior vena cava syndrome, New York Heart Association (NYHA) classification of heart disease ≥2 within 3 months before entry, or presence of cardiac disease that in the opinion of the Investigator increases the risk of ventricular arrhythmia.
11. Pregnancy or nursing (women of child-bearing potential).
12. Patients suffering from diseases with an increased chance of liver toxicity.
13. Patients suffering from psychic disorders that make a comprehensive judgement impossible, such as psychosis, hallucinations and/or depression.
14. Patients who are declared incompetent.
15. Previous enrolment in the present study or previous treatment with SIRT.
16. Female patients who are not using an acceptable method of contraception (oral contraceptives, barrier methods, approved contraceptive implant, long-term injectable contraception, intrauterine device or tubal ligation) OR are less than 1 year postmenopausal or surgically sterile during their participation in this study (from the time they sign the consent form) to prevent pregnancy.
17. Male patients who are not surgically sterile or do not use an acceptable method of contraception during their participation in this study (from the time they sign the consent form) to prevent pregnancy in a partner.
18. Patients with abnormalities of the bile ducts (such as stents) with an increased chance of infections of the bile ducts (papillotomy and cholecystectomy are allowed). Or evidence of extensive portal hypertension, splenomegaly, ascites or active hepatitis (B and/or C).
19. Body weight over 150 kg.
20. Severe allergy for i.v. contrast (Visipaque®), used for CT evaluation, pre-treatment angiography and treatment angiography.
21. Liver tumour involvement ≥70% as quantified on CT

***Matched Pair Analysis data management***

An independent data monitor was appointed, who supported study execution and checked data consistency (Clinitude CRO, Belgium). Demographic and clinical data was provided by the local investigators into an electronic case report form (eCRF, Caster EDX). All imaging provided by the centers was pseudonymized and securely transferred to the central review site (UMC Utrecht, the Netherlands). All CT/MRI imaging (baseline and all follow-up imaging studies) were blinded and re-assessed according to RECIST 1.1 by the central reviewers (WBV and AJATB; >10 years’ experience) for both cohorts. Fractional liver tumor involvement (tumor volume divided by total liver volume) was acquired by segmenting the provided CT using a convolutional neural network (Quantib B.V., the Netherlands). All segmentations were manually checked and corrected by the central reviewers. On MRI, this was manually segmented in Syngo.via (Siemens, Erlangen) by the central reviewers.

#### **Supplemental data 2**

Match propensity score parameters and categories considered in the model:

*Prior to [^177^Lu]Lu-PRRT*

- Age (e.g. categorized as <=50, 51-60, 61-70, >=70)
- Gender (male/female)
- Neuroendocrine neoplasm origin (Pancreas, Small Intestine, Bronchus/Lung, or Other)
- KI67 score (e.g. 0-2%; 3-5%, 5-10% and 10-20%)
- Relative days between first diagnosis of the disease and start of PRRT treatment (e.g.<= 3 years, > 3 years)
- Previous resection of the primary tumour (Y/N)
- Previous Somatostatin analogues (SSA) (Y/N)
- Previous Sunitinib/Everolimus (Y/N)
- Previous chemotherapy (Y/N)
- Previous bland-embolization or TACE (Y/N)

*3±1 month after final [^177^Lu]Lu-PRRT*

- Eastern Cooperative Oncology Group (ECOG) performance score (e.g. ≤1, >1)
- Albumin level (<ULN, normal)
- Fractional liver involvement at 3±1 month after last cycle of PRRT (e.g. 0-10%; 10-25%, 25-50%; >50%), as determined by central review
- Extrahepatic disease (Y/N)
- Concurrent Somatostatin analogues use (i.e. prior or subsequent to PRRT or ^166^Ho-SIRT treatment) (Y/N)
- Objective response according to RECIST 1.1 [Partial Response (PR), Stable Disease (SD), Progressive Disease (PD)], as determined by central review.

**Supplemental data 3**

*Details on full analysis set data*

The observed differences in MPA were also noted in FAS. PFS_2y_ was higher for PRRT+^166^Ho-SIRT cohort, 67% (95% CI 43%-85%) versus 52% (95% CI 40%-64%) in PRRT-only cohort. Also hPFS_2y_, was higher in the PRRT+^166^Ho-SIRT cohort (79%; 95% CI 54%-94%) versus PRRT-only (53%; 95% CI 41%-65%). No significant differences in median PFS and hPFS were observed

PFS in the PRRT+^166^Ho-SIRT cohort was 30.7 months (95% CI 25.4-NE) and 32.9 months (95% CI 26.8-NE) in the PRRT-only cohort. Of note, in the PRRT+^166^Ho-SIRT cohort, 62% (16/26) had events, of which 42% (11/26) had progressive disease and 19% (5/26) had death as an event. In the PRRT-only cohort, 42% (31/74) had events; all were progressive disease events. Similar to the MPA, in the gastrointestinal subgroup, a temporary delay in time to progression or death in the PRRT+^166^Ho-SIRT cohort was found (see figure below).


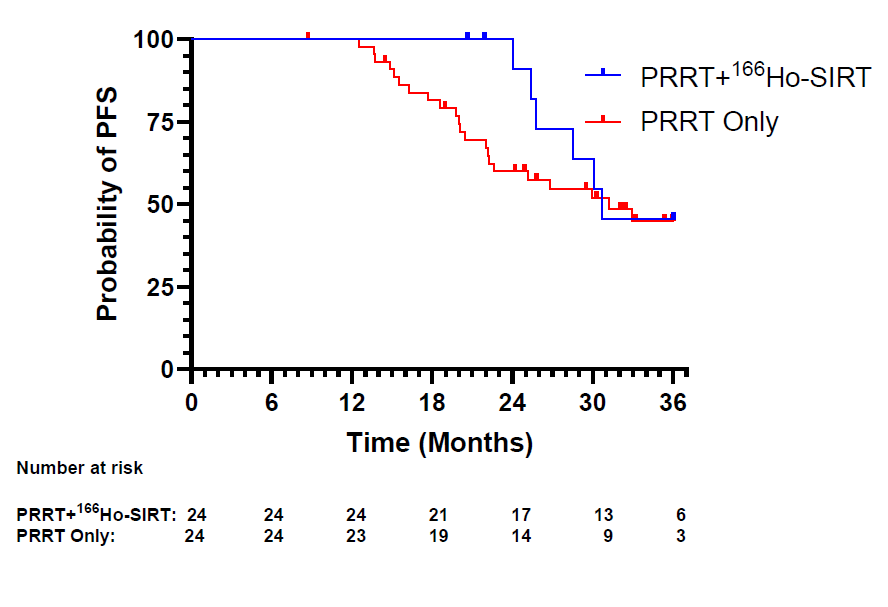


Median hPFS was 31.2 months (95% CI 25.8-NE) in the PRRT+^166^Ho-SIRT cohort and 32.9 (95% CI 28.5-NE) in the PRRT-only cohort. In the PRRT+^166^Ho-SIRT cohort, of the 52% (13/25) patients with events, 28% (7/25) had progressive disease and 24% (6/25) had death as event. In the PRRT-only arm in FAS, 41% (32/78) had an event of which 39.7% (31/78) had progressive disease as event and one death (1.3%). In line with PFS, the temporary delay in hepatic progression or death was more pronounced in the gastrointestinal subgroup (see figure below).

ORR was also confirmed to be higher; PRRT+^166^Ho-SIRT 79% (95% CI 54%-94%) versus PRRT-only 53% (95% CI 41%-65%). Overall, bORR was considerably higher in the PRRT+^166^Ho-SIRT cohort than the PRRT-only cohort (70% vs 13%); this is also reflected by intrahepatic bORR (77% vs 16%). Also in FAS, the localized hepatic effect of ^166^Ho-SIRT is underscored by absence of bORR improvement in the extrahepatic radiological response (FAS: 13% vs 9%).

Finally, median time to subsequent treatment was 36.6 months (95% CI 33.7-NE) in the PRRT+^166^Ho-SIRT cohort and 32.4 months (95% CI 29.8-36.0) in the PRRT-only cohort.


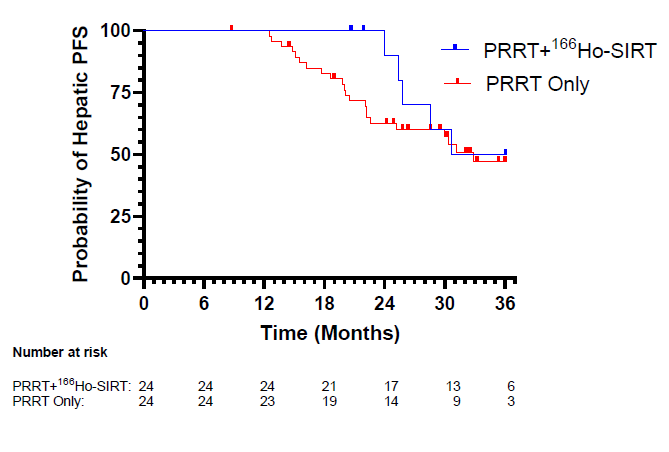


#### **Supplemental Table 1**

#### ‘Supplemental Table 1. Collected data and time points for analysis’

| **Data collection** | **Baseline (pre PRRT)** | **PRRT** | **3±1 month after last PRRT cycle** | **^166^Ho-SIRT** | **6 to 24**  **month Follow up**** |
| --- | --- | --- | --- | --- | --- |
| Demographics* | × |  |  |  |  |
| Patient Status (ECOG PS) | × |  | × |  |  |
| Previous treatments  (i.e. surgical, local and systemic) | × |  |  |  |  |
| Laboratory investigations | × |  | × |  |  |
| PRRT^†^ |  | × |  |  |  |
| ^166^Ho-SIRT |  |  |  | × |  |
| Anatomical Imaging | × |  | × |  | × |
| Toxicity data (adverse events and clinical hormone-related complaints by NET) | × |  | × |  | × |

*Gender, age, time of NET diagnosis, primary NET origin, WHO grade, Ki67-index primary, time of liver metastases development, metastases WHO grade, metastases Ki67-index)

^†^Activity per cycle, cumulative activity, interval between cycles, total treatment time.

**6 months after last PRRT cycles in PRRT-only cohort. 3 months after ^166^Ho-SIRT (≈6 months after last PRRT cycle) in the PRRT+^166^Ho-SIRT cohort.

**Supplemental Table 2. Best objective response rate during follow-up**

|  | **Full Analysis Set** | | **Matched Analysis Set** | |
| --- | --- | --- | --- | --- |
| **Label** | **PRRT+^166^Ho-SIRT**  **(N=30)** | **PRRT Only**  **(N=90)** | **PRRT+^166^Ho-SIRT**  **(N=24)** | **PRRT Only**  **(N=24)** |
| **Best general radiological response** | | | | |
| CR | 0% [0 / 30] | 0% [0 / 90] | 0% [0 / 24] | 0% [0 / 24] |
| PR | 70.0% [21 / 30] | 13.3% [12 / 90] | 70.8% [17 / 24] | 25.0% [6 / 24] |
| SD | 26.7% [8 / 30] | 71.1% [64 / 90] | 29.2% [7 / 24] | 75.0% [18 / 24] |
| PD | 3.3% [1 / 30] | 15.6% [14 / 90] | 0% [0 / 24] | 0% [0 / 24] |
| **Best intrahepatic radiological response** | | | | |
| CR | 3.3% [1 / 30] | 0.0% [0 / 90] | 4.2% [1 / 24] | 0.0% [0 / 24] |
| PR | 73.3% [22 / 30] | 15.6% [14 / 90] | 70.8% [17 / 24] | 29.2% [7 / 24] |
| SD | 23.3% [7 / 30] | 72.2% [65 / 90] | 25.0% [6 / 24] | 70.8% [17 / 24] |
| PD | 0.0% [0 / 30] | 12.2% [11 / 90] | 0% [0 / 24] | 0% [0 / 24] |
| **Best extrahepatic radiological response** | | | | |
| CR | 0% [0 / 30] | 0% [0 / 90] | 0% [0 / 24] | 0% [0 / 24] |
| PR | 13.3% [4 / 30] | 8.9% [8 / 90] | 16.7% [4 / 24] | 12.5% [3 / 24] |
| SD | 70.0% [21 / 30] | 48.9% [44 / 90] | 70.8% [17 / 24] | 75.0% [18 / 24] |
| PD | 10.0% [3 / 30] | 8.9% [8 / 90] | 4.2% [1 / 24] | 0.0% [0 / 24] |
| NA/Unknown | 6.7% [2 / 30] | 33.3% [30 / 90] | 8.3% [2 / 24] | 12.5% [3 / 24] |

**Supplemental table 3. PRRT details and imaging results 3 months after PRRT**

|  | **Full Analysis Set** | | **Matched Analysis Set** | |
| --- | --- | --- | --- | --- |
| **Label** | **PRRT+^166^Ho-SIRT**  **(N=30)** | **PRRT Only**  **(N=90)** | **PRRT+^166^Ho-SIRT**  **(N=24)** | **PRRT Only**  **(N=24)** |
| **PRRT** | Mean ± SD | Mean ± SD | Mean ± SD | Mean ± SD |
| Total Duration of PRRT (months) | 6.60±1.07 | 6.18±0.94 | 6.61±1.05 | 6.15±0.89 |
| Cumulative activity of PRRT (GBq) | 29.72±0.27 | 29.76±0.88 | 29.68±0.27 | 29.67±1.01 |
| **IMAGING RESULTS 3 MONTHS AFTER PRRT** | |  |  |  |
| Median Liver Tumor Burden in % (range) | 7, (1; 84) | 12, (0; 81) | 6, (1; 84) | 13, (0; 69) |
| **LIVER TUMOR DISTRIBUTION** |  |  |  |  |
| Uni-Lobar | 6.7% | 7.8% | 8.3% | 4.2% |
| Bi-Lobar | 93.3% | 92.2% | 91.7% | 95.8% |
| **PATTERN OF LIVER DISEASE** |  |  |  |  |
| Simple | 3.3% | 3.3% | 4.2% | 0% |
| Complex | 3.3% | 8.9% | 4.2% | 8.3% |
| Diffuse | 93.3% | 87.8% | 91.7% | 91.7% |
| **OBJECTIVE RESPONSE AFTER PRRT ALONE*** | | | | |
| Partial response | 27% | 9% | 25% | 17% |
| Stable disease | 60% | 73% | 75% | 83% |
| Progressive disease | 13% | 18% | 0% | 0% |
| **EXTRAHEPATIC DISEASE** |  |  |  |  |
| Extrahepatic Disease | 83.3% | 64.4% | 87.5% | 87.5% |
| Lymph Nodes | 50% | 50% | 58.3% | 58.3% |
| Bones | 13.3% | 24.4% | 12.5% | 37.5% |
| Lungs | 20% | 6.7% | 25% | 8.3% |
| Pleuritis | 0% | 0% | 0% | 0% |
| Peritonitis | 6.7% | 0% | 4.2% | 0% |
| Other | 33.3% | 21.1% | 33.3% | 33.3% |

**Supplemental table 4. Differences in Eastern Cooperative Oncology Group Performance scores, comparison to FAS**

|  | **Full Analysis Set** | | **Matched Analysis Set** | | |
| --- | --- | --- | --- | --- | --- |
|  | **PRRT+^166^Ho-SIRT**  **(N=30)** | **PRRT Only**  **(N=90)** | **PRRT+^166^Ho-SIRT**  **(N=24)** | | **PRRT Only**  **(N=24)** |
| **ECOG BEFORE PRRT** |  |  |  | |  |
| 0 | 56.7% | 65.6% | 50% | | 58.3% |
| 1 | 40% | 31.1% | 45.8% | | 37.5% |
| 2 | 3.3% | 3.3% | 4.2% | | 4.2% |
| **ECOG 3 MONTHS AFTER PRRT ALONE*** |  |  |  |  | |
| 0 | 0% | 68.9% | 0% | | 75% |
| 1 | 63.3% | 31.1% | 58.3% | | 25% |
| 2 | 33.3% | 0% | 37.5% | | 0% |
| 3 | 3.3% | 0% | 4.2% | | 0% |

**Supplemental Table 5. Subsequent treatments**

| **Label** | **Full Analysis Set** | | **Matched Analysis Set** | |
| --- | --- | --- | --- | --- |
|  | **PRRT+^166^Ho-SIRT**  **(N=30)** | **PRRT Only**  **(N=90)** | **PRRT+^166^Ho-SIRT**  **(N=24)** | **PRRT Only**  **(N=24)** |
|  | % [n/N] | % [n/N] | % [n/N] | % [n/N] |
| SSA | 10.0% [3 / 30] | 6.7% [6 / 90] | 8.3% [2 / 24] | 8.3% [2 / 24] |
| Chemotherapy | 3.3% [1 / 30] | 20.0% [18 / 90] | 4.2% [1 / 24] | 12.5% [3 / 24] |
| Bland Embolization | 0.0% [0 / 30] | 10.0% [9 / 90] | 0.0% [0 / 24] | 8.3% [2 / 24] |
| Everolimus / Sunitinib | 6.7% [2 / 30] | 11.1% [10 / 90] | 4.2% [1 / 24] | 12.5% [3 / 24] |
| TACE | 0.0% [0 / 30] | 4.4% [4 / 90] | 0.0% [0 / 24] | 4.2% [1 / 24] |
| TARE | 3.3% [1 / 30] | 7.8% [7 / 90] | 4.2% [1 / 24] | 8.3% [2 / 24] |
| Additional Surgery | 3.3% [1 / 30] | 2.2% [2 / 90] | 4.2% [1 / 24] | 0.0% [0 / 24] |
| Re-PRRT | 36.7% [11 / 30] | 42.2% [38 / 90] | 37.5% [9 / 24] | 54.2% [13 / 24] |
| Other | 6.7% [2 / 30] | 14.4% [13 / 90] | 8.3% [2 / 24] | 16.7% [4 / 24] |
| None | 56.7% [17 / 30] | 31.1% [28 / 90] | 58.3% [14 / 24] | 12.5% [3 / 24] |
